# Positive impact of Platelet-rich plasma and Platelet-rich fibrin on viability, migration and proliferation of osteoblasts and fibroblasts treated with zoledronic acid

**DOI:** 10.1038/s41598-019-43798-z

**Published:** 2019-06-05

**Authors:** Daniel Steller, Nele Herbst, Ralph Pries, David Juhl, Samer G. Hakim

**Affiliations:** 1 University Hospital Schleswig-Holstein, Department of Maxillofacial Surgery, Ratzeburger Allee 160, 23562 Luebeck, Germany; 2 University Hospital Schleswig-Holstein, Department of Otolaryngology-Head and Neck Surgery, Ratzeburger Allee 160, 23562 Luebeck, Germany; 3 University Hospital Schleswig-Holstein, Department of Transfusion Medicine, Ratzeburger Allee 160, 23562 Luebeck, Germany

**Keywords:** Medical research, Cell biology

## Abstract

Bisphosphonates are frequently used for the antiresorptive treatment in bone metastasis diseases or for osteoporosis. A side effect of this therapy is osteonecrosis of the jaw. This inhibits osteoclast function, but osteoblasts and fibroblasts are also negatively affected in terms of impaired proliferation. Additive local treatment with platelet-rich plasma (PRP) and platelet-rich fibrin (PRF) promotes adhesion, proliferation and migration of cells due to high concentrations of growth factors like PDGF, TGF and IGF. The aim of the study was to investigate the effect of PRP or PRF on proliferation, migration and viability of osteoblasts and oral fibroblasts, treated with zoledronic acid (ZA). ZA treated fibroblasts and osteoblasts were exposed to PRP/PRF. Cell proliferation, migration and viability were measured using the real-time cell-analyzer assay (RTCA), the scratch assay and the MTT assay. There was a significant increase in closure of the scratch area by PRP/PRF treated osteoblasts (PRP = 40.6%, PRF = 100.0%, NC = 0.0%) as well as fibroblasts (PRP = 100.0%, PRF = 100.0%, NC = 12.7%) in comparison to the group of negative control (all *p* ≤ *0*.*05*). Furthermore, the negative effect of ZA on cell migration was generally reduced in both cell lines using PRP/PRF. The viability and proliferation of cells decreased after exposure to ZA, whereas we observed an enhancement of cell viability within 24 hours by application of PRP/PRF in ZA treated cells. The negative effect of ZA on cell proliferation was especially reduced when using PRF. The use of PRF/PRP improves the behavior of ZA-treated cells, but PRF appears to have an advantage in comparison to PRP. This study demonstrates that treatment with PRF/PRP may have positive effects in the therapy of Bisphosphonate-Related Osteonecrosis of the Jaw (BRONJ).

## Introduction

Bisphosphonates are widely used for the treatment of abnormal bone metabolism frequently found in bone metastasis or in osteoporosis. Zoledronic acid (ZA) belongs to the third generation of bisphosphonates and has been shown to reduce proliferation and viability of tumor cells and even improve the overall survival of cancer patients beyond the prevention of bone resorption^1,2^. The presence of nitrogen in its molecular structure leads to a stronger inhibition of osteoclast activity than seen in other bisphosphonates^3^. Therefore, and because of the increasing use of bisphosphonates, there has been an increase in one of its major side effects, osteonecrosis in the jaw (BRONJ)^4^. Bisphosphonate-associated bone necrosis is defined as a disease with exposed bone in the maxilla or mandible, lack of healing of bony lesion for more than 8 weeks and when other risk factors (e.g. radiotherapy) are ruled out no oral radiotherapy history^5^. The occurrence of BRONJ depends in particular on the type, duration and form of ZA administration^6–8^. It is known that ZA has also cytotoxic effects on osteoblasts and leads to inhibition of proliferation, adhesion and cell migration^9,10^. Further fibroblasts are, affected by bisphosphonate therapy as well. ZA blocks their growth and reduces the transcription of type I collagen in human fibroblasts in a dose-dependent manner. This is of crucial importance in wound healing process^11^. The treatment of BRONJ is often resistant to conventional surgical debridement and recurrence occurs frequently (11–29%^12^). An interruption or discontinuation of bisphosphonate medication (drug holidays) does not lead to the desired therapeutic effect^13^. In order to improve wound healing and reduce the rate of recurrence, local therapeutic measures are becoming increasingly popular. These are based on faster adhesion of soft tissue to the affected bone areas, e.g. surgical debridement combined with local application of platelet-rich blood products^14^. Various studies have shown that both platelet-rich plasma (PRP) and platelet-rich fibrin (PRF) promote settlement, adhesion, proliferation and migration of osteoblasts due to high concentrations of growth factors like PDGF, TGF and IGF leading to improved wound healing^15,16^. For example, PDGF is a powerful stimulus for the chemoattraction of human bone-derived osteoblasts at various stages of differentiation^17^, TGF-β1 is a regulatory protein involved in bone remodelling and fracture healing^18^, and VEGF supports bone healing by promoting vascular structures^19^. In a previous study, the content of both PRP and PRF used in the present study was analysed and it was shown that leucocytes depleted PRP contains a reasonably higher concentration of PDGF and TGF-β than PRF^20^.

The aim of this study was to investigate the impact of PRP or PRF on proliferation, migration and viability of bisphosphonate-treated osteoblasts and oral fibroblasts in an effect to enhance local treatment of BRONJ.

## Results

### Preinvestigation for determination of optimal PRP and PRF concentration

**Figure 1:**
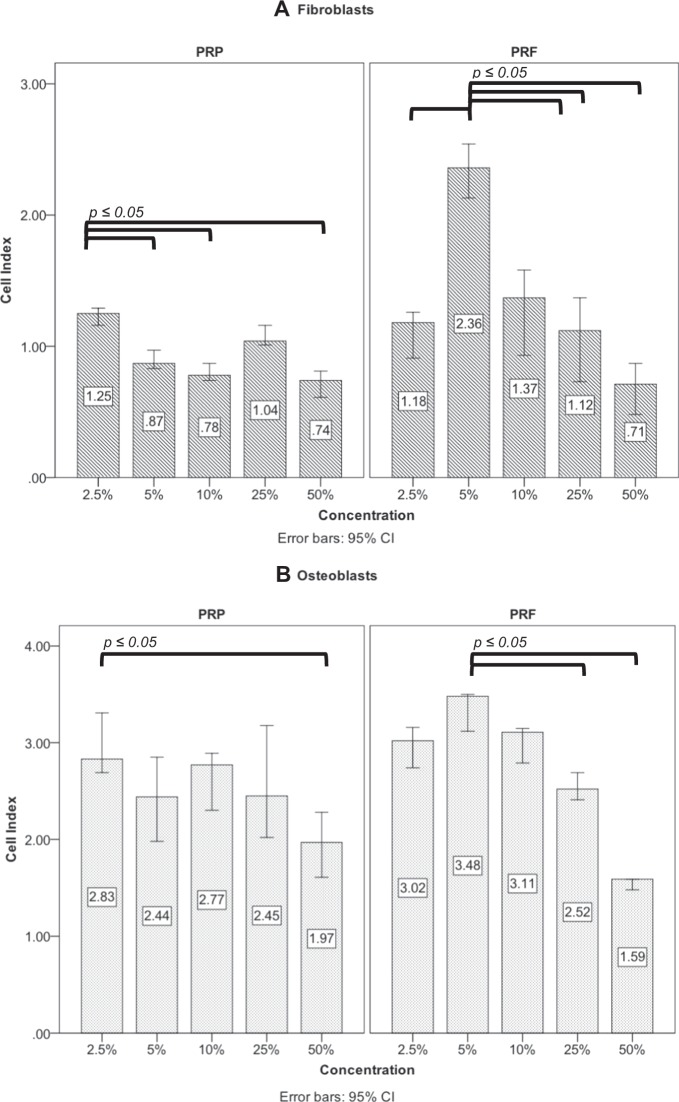
Preinvestigation of PRP and PRF for *in-vitro* addition. The effect of PRP and PRF were examined in different concentrations to fibroblasts (**A**) and osteoblasts (**B**) by real- time cell analysis.

The RTCA assay was performed with different dilutions of PRP and PRF. With regard to PRP, the dilution of 2.5% showed a significant increase in proliferation of fibroblasts compared to the other dilutions (Fig. 1A). For osteoblasts proliferation, the 2.5% concentration also showed the highest increase in comparison to 5%, 10% as well as 25% (Fig. 1B). Concerning PRF, the concentration of 5% PRF showed significant increase in proliferation of fibroblasts compared to 2.5%, 10%, 25% and 50% (*p* ≤ *0*.*05*, each). The Osteoblast proliferation mostly increased using 5%, too (Fig. 1B).

### Scratch assay

**Figure 2:**
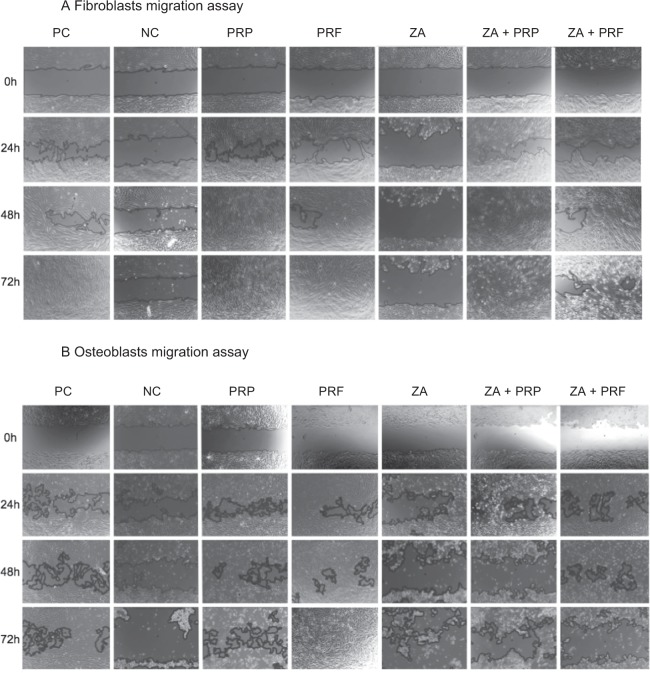
Representative photographs of cell migration using the scratch assay. The effect of ZA, PRP, PRF and their combination on the migration of fibroblasts (**A**) and osteoblasts (**B**) were examined (PC = positive control; NC = negative control; ZA = zoledronic acid).

**Figure 3:**
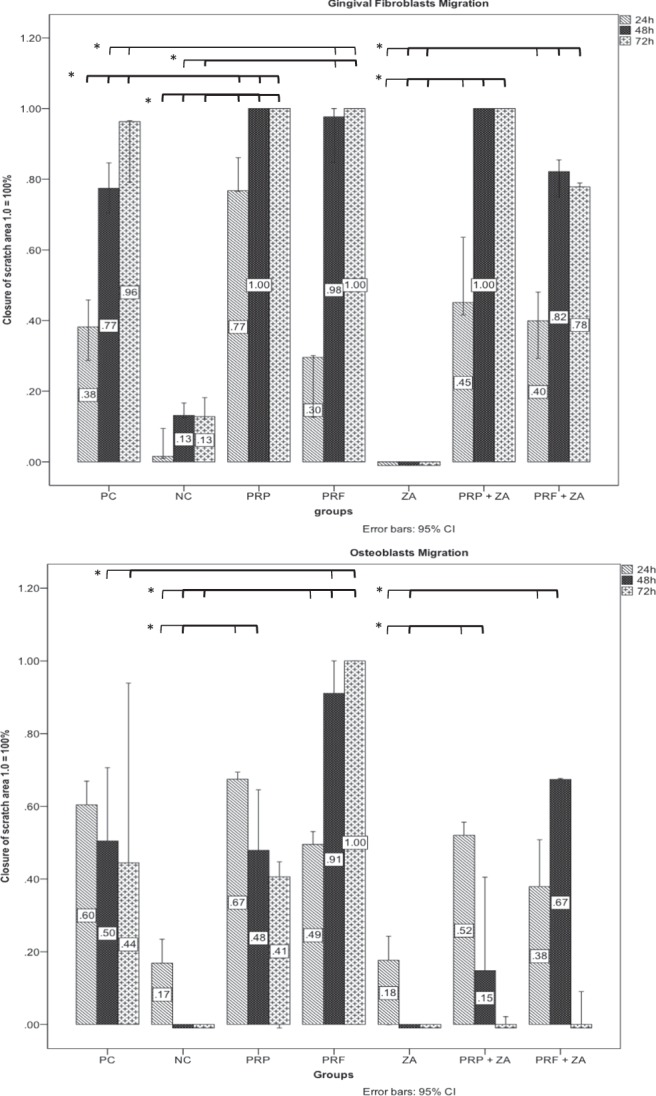
Percentage of closure of the scratch area in fibroblasts (**A**) and osteoblasts (**B**) in the different groups (1.0 = 100% coverage). Significant difference (*p* < 0.05) is marked with *.

Closure of the scratch defect was defined as 100% cell confluence. Human gingival fibroblasts showed closure of the scratch area in the group of positive control (manufacture recommended medium with the use of 10% fetal bovine serum) within 72 h. With reduction of the fetal bovine serum (FBS) to 1% in the group of negative control, it was not possible to observe closure of scratch defect in both fibroblasts and osteoblasts, anymore (Fig. 2). ZA application to fibroblasts as well as osteoblasts did not show any cell migration within 72 h observation-time, too. Fibroblasts treated by PRF showed closure of the scratch defect within 72 h (Figs 2, 3 and Table 1). At the 48 h measurement, there was already a significant increase of cell migration by PRF (97.6; CI: 73.8–100.0; *p* ≤ 0.05) in comparison to the positive and negative control groups (77.4; CI: 60.0–95.0 and 13.1; CI: 8.5–19.6, respectively). Cell stimulation by PRP led to a significant increase in closure of the scratch area in comparison to both control groups (*p* ≤ 0.05), too. Cells exposed to PRP reached 100% confluence within 48 h, even in the group containing ZA. After adding platelet derivatives (PRP or PRF) and ZA simultaneously, a significant increase in scratch area closure was observed at each time point in comparison to ZA alone (*p* ≤ 0.05) (Figs 2A and 3A). The osteoblasts (Figs 2B, 3B and Table 1) showed complete closure of the scratch within 72 h only in the PRF group. Therefore, PRF led to a significant increase in migration in comparison to PC and NC at 48 h (*p* ≤ 0.05). Neither of the other groups showed closure of the scratch defects within 72 h. Furthermore, the addition of PRP or PRF to ZA improved cell migration and inhibited cell impairment by ZA within the first 48 h - (cell confluence = 52.0% (CI: 47.9–58.4) in the PRP + ZA, 37.9% (CI: 17.4–63.6) in the PRF + ZA at 24 h, 14.8% (CI: 0.0–61.5) in the PRP + ZA (*p* = 0.225) and 67.4% (CI: 59.7–72.5) in the PRF + ZA at 48 h; each *p* ≤ 0.05). Figure 3B and Table 1 summarizes the scratch assay data.

**Table 1:** Migration of fibroblasts and osteoblasts using the scratch assay.

Migration – assay Fibroblasts	24 h	48 h	72 h
PC	*38.2 (CI: 16.1–59.0)	*77.4 (CI: 60.0–95.0)	*96.3 (CI: 65.8–100.0)
NC	1.5 (CI: 0.0–15.7)	13.1 (CI: 8.5–19.6)	12.7 (CI: 6.0–22.6)
PRP	*76.7 (CI: 66.2–93.3)	*100.0	*100.0
PRF	*29.6 (CI: 0.0–48.8)	*97.6 (CI: 73.8–100.0)	*100.0
ZA	0.0	0.0	0.0
ZA + PRP	*45.1 (CI: 20.7–79.4)	*100.0	*100.0
ZA + PRF	*39.9 (CI: 15.7–62.5)	*82.1 (CI: 67.6–94.2)	*77.8 (CI: 76.5–79.8)
**Migration – assay Osteoblasts**	**24** **h**	**48** **h**	**72** **h**
PC	*60.4 (CI: 8.6–100.0)	*50.5 (CI: 16.9–91.4)	*44.4 (CI: 0.0–100.0)
NC	16.9 (CI: 0.0–35.8)	0.0	0.0
PRP	*67.5 (CI: 26.7–94.5)	*47.9 (CI: 0.0–94.3)	40.6 (CI: 0.0–90.9)
PRF	*49.5 (CI: 42.6–57.2)	*91.1 (CI: 73.4–100.0)	*100.0
ZA	17.7 (CI: 0.0–45.2)	0.0	0.0
ZA + PRP	*52.0 (CI: 47.9–58.4)	14.8 (CI: 0.0–61.5)	0.1 (CI: 0.0–4.6)
ZA + PRF	*37.9 (CI: 17.4–63.6)	*67.4 (CI: 59.7–72.5)	0.1 (CI: 0.0–16.8)

(PC = positive control; NC = negative control, PRP = platelet-rich plasma; PRF = platelet-rich fibrin, ZA = zoledronic acid) values are medians of 3 replicates in percent of scratch closure and confindence intervall; * = significant results compared to NC (negative control).

### MTT assay

**Figure 4:**
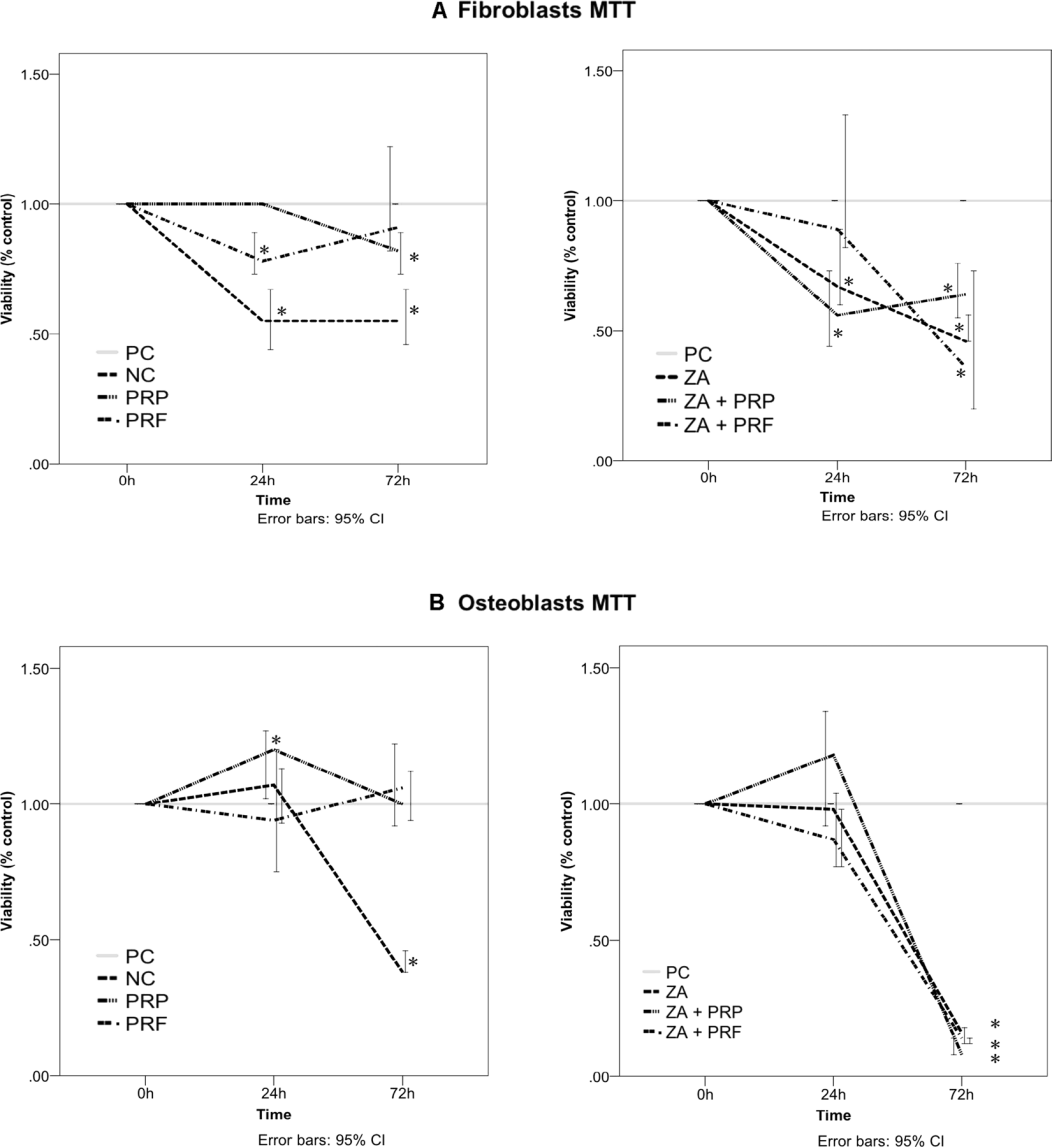
Effects of PRP, PRF, ZA and their combinations on the viability of human gingival fibroblasts (**A**) and osteoblasts (**B**) assessed by MTT assay. Significant difference (*p* < 0.05) is marked with *.

The positive control, PRP and PRF groups showed a comparable effect on the viability of fibroblasts during the observation time period (Fig. 4A). Using ZA (ZA group), we observed cytotoxic effects on fibroblasts after 24 h (ZA = 67.0% (CI: 60.0–89.0%) vs. PC = 100%; *p* ≤ 0.05)) (Fig. 4A and Table 2). Furthermore, we found a positive effect of PRF on the viability of ZA treated fibroblasts (Fig. 4A). In the PRF + ZA group, cell viability increased in comparison to the ZA group within 24 h (PC = 100.0% vs. ZA + PRF = 89.0% (CI: 82.0–133.0%) *p* = *0*,*49*; PC = 100.0% vs. ZA = 67.0% (CI: 60.0–89.0%) *p* ≤ 0.05), afterwards, the cytotoxic effect of ZA predominated in all groups. Osteoblasts treated with PRP and PRF also showed a comparable effect to PC (Fig. 4B, Table 2). After 72 h the cytotoxic effect of ZA predominated also in osteoblasts culture regardless of the PRP/PRF addition. (PC = 100.0 ± 0.0%; ZA = 16.0% (CI: 12.0–18.0%); ZA + PRP = 8.0% (CI: 8.0–14.0%); ZA + PRF = 14.0% (CI: 12.0–14.0%) each in comparison to PC *p* ≤ 0.05; Fig. 4B, Table 2).

**Table 2:** Results of the MTT test performed on fibroblasts and osteoblasts at different times.

MTT - Assay Fibroblasts	24 h	72 h
NC	*55.0 (CI: 44.0–67.0)	*55.0 (CI: 46.0–67.0)
PRP	100.0	*82.0 (CI: 73.0–89.0)
PRF	*78.0 (CI: 73.0–89.0)	91.0 (CI: 82.0–122.0)
ZA	*67.0 (CI: 60.0–89.0)	*46.0 (CI: 46.0–56.0)
ZA + PRP	*56.0 (CI 44.0–73.0)	*64.0 (CI: 55.0–76.0)
ZA + PRF	89.0 (CI: 82.0–133.0)	*36.0 (CI: 20.0–73.0)
**MTT - Assay Osteoblasts**	**24 h**	**72 h**
NC	100.7 (CI: 75.0–120.0)	*38.0 (CI: 38.0–46.0)
PRP	*120.0 (CI: 102.0–127.0)	100.0 (CI: 92.0–122.0)
PRF	94.0 (CI: 93.0–113.0)	106 (CI: 94.0–112.0).
ZA	98.0 (CI: 77.0–104.0)	*16.0 (CI: 12.0–18.0)
ZA + PRP	118.0 (CI: 0: 92.0–134.0)	*8.0 (CI: 8.0–14.0)
ZA + PRF	87.0 (CI: 77.0–98.0)	*14.0 (CI: 12.0–14.0)

(PC = positive control; NC = negative control, PRP = platelet-rich plasma; PRF = platelet-rich fibrin, ZA = zoledronic acid); values are medians in percent of positive control and 95.0% confidence interval * = Significant result compared to PC (positive control).

### Real time cell analysis assay

**Figure 5:**
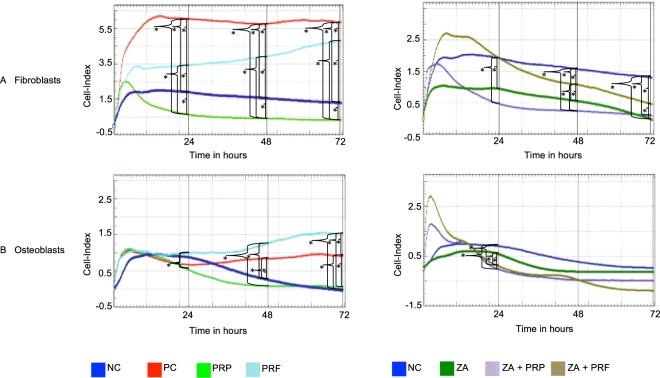
Cell proliferation assessed by real-time cell analysis for fibroblasts (**A**) and osteoblasts (**B**) in the different groups over a period of 72 h. The values are the median of the measured cell index. Significant difference (*p* < 0.05) is marked with *.

The proliferation of fibroblasts and osteoblasts was examined by real-time cell analysis (Fig. 5). PRF induced a significant increase in the proliferation of both fibroblasts and osteoblasts at 72 h compared to the negative control and PRP (Table 3, Fig. 5). PRF led to a fibroblast proliferation cell index of 4.86 (CI: 4.56–5.07) compared to 1.25 (CI: 0.96–1.63) for the NC (*p* ≤ 0.05) or 0.34 (CI: 0.0–0.67) with PRP (*p* ≤ 0.05) at 72 h. PRF further increased the proliferation of osteoblasts to 1.20 (CI: 0.23–2.62), compared to 0.24 (CI: 0.0–1.06) in the NC (*p* ≤ 0.05) or 0.15 (CI: 0.0–0.42) with PRP (*p* ≤ 0.05). PRP had no effect on the proliferation of fibroblasts and osteoblasts (Table 3 and Fig. 5). Exposure of fibroblasts and osteoblasts to ZA led to a significant reduction in proliferation throughout the study period (Table 3 and Fig. 5) (ZA = 0.0 vs. PC = 5.83 (CI: 5.20–6.49); *p* ≤ 0.05 and ZA = 0.0 vs. PC = 0.98 (CI: 0.80–1.13); *p* ≤ 0.05, respectively). If PRF was added along with ZA, the proliferation of fibroblasts but not of osteoblasts significantly increased compared to the ZA group (ZA + PRF = 0.42 (CI: 0.06–0.88) vs. ZA = 0.0; *p* ≤ 0.05).

**Table 3:** cell proliferation of fibroblasts and osteoblasts using the Real-time cell analysis assay.

RTCA - Fibroblasts	24 h	48 h	72 h
PC	6.03 (CI: 5.91–6.14)	5.77 (CI: 5.32–6.21)	5.83 (CI: 5.20–6.49)
NC	1.89 (CI: 1.55–2.24)	1.54 (CI: 1.16–1.94)	1.25 (CI: 0.96–1.63)
PRP	0.63 (CI: 0.51–0.73)	0.43 (CI: 0.12–0.72)	0.34 (CI: 0.00–0.67)
PRF	3.66 (CI: 2.21–4.72)	3.95 (CI: 3.80–4.17)	4.86 (CI: 4.56–5.07)
ZA	0.95 (CI: 0.83–1.04)	0.53 (CI: 0.45–0.63)	0.0 ± 0.0
ZA + PRP	0.42 (CI: 0.32–0.57)	0.23 (CI: 0.14–0.34)	0.09 (CI: 0.03–0.15)
ZA + PRF	1.86 (CI: 1.08–2.67)	0.96 (CI: 0.39–1.73)	0.42 (CI: 0.06–0.88)
**RTCA - Osteoblasts**	**24** **h**	**48** **h**	**72** **h**
PC	0.69 (CI: 0.47–0.86)	0.87 (CI: 0.68–1.00)	0.98 (CI: 0.80–1.13)
NC	0.92 (CI: 0.59–1.14)	0.23 (CI: 0.19–0.29)	0.24 (CI: 0.00–1.06)
PRP	0.49 (CI: 0.42–0.56)	0.09 (CI: 0.01–0.16)	0.15 (CI: 0.00–0.42)
PRF	0.86 (CI: 0.17–1.75)	1.01 (CI: 0.34–2.03)	1.20 (CI: 0.23–2.62)
ZA	0.47 (CI: 0.29–0.75)	0.0 ± 0.0	0.0 ± 0.0
ZA + PRP	0.0 ± 0.0	0.0 ± 0.0	0.0 ± 0.0
ZA + PRF	0.06 (CI: 0.0–0.28)	0.0 ± 0.0	0.0 ± 0.0

(PC = positive control; NC = negative control, PRP = platelet rich plasma; PRF = platelet rich fibrin, ZA = zoledronic acid); CI = confindence interval of 95%; values are medians of the cell index measured by RTCA.

## Discussion

Platelet-enriched blood derivatives (PRP/PRF) provide a therapeutic tool for promoting tissue repair. Some studies have demonstrated that both support cell proliferation and osteogenic differentiation as well as development^21–24^. In this study, we show the influence of PRP and PRF on ZA-treated osteoblasts and oral fibroblasts. In order to explain the effect of these preparations on osteoblasts treated with ZA, it is necessary to identify the interaction of osteoblasts with ZA. The effect of ZA on osteoblasts depends on the concentrations used. On one hand, it can prolong the lifespan of osteoblasts using lower concentrations (10^−9^ M to 10^−6^ M)^19,25^, on the other hand it can lead to apoptosis at concentrations above 10^−5^ M^26^. Following hypotheses have been postulated to explain the mechanisms underlying these effects in osteoblasts. Some studies suggested that ZA interferes with the metabolism of osteoblasts by modulating Connexin 43 (Cx43)^27^. TGF-β1 as a main component of PRP - and to a lesser extent of PRF- is also known to alter the expression of Cx43^28^ and therefore, both PRP and PRF may in turn modulate the effect of ZA on osteoblasts. More recently, it has also been shown that protein tyrosine phosphatases (PTPs) may represent one ZA target in osteoblasts and that this interaction leads to osteoblast proliferation, modulation of cytosolic Ca2+ levels, their maturation and differentiation^25^. Further, it is known that a variety of ion channels play a leading role in the regulation of bone homeostasis, including K+ channels and transient receptor potential (TRP) channels^29^. PRF and PRP may act against ZA-induced bone loss by modulating those ion channels, farnesylpyrophosphate (hFPPS) as well as geranyl-geranylpyrophosphate synthase (hGGPPS)^30^.

We assessed an enhancement of fibroblast and osteoblast migration by both PRP and PRF within 72 h in comparison to the groups of positive and negative control. PRP better promoted fibroblast migration than osteoblasts’ and seems to have a higher impact of soft tissue regeneration. This is in accordance with a recent study by Kobayashi *et al*. who proved this effect by improved expression of extracellular matrix in fibroblast compared to osteoblasts^31^. Similar studies reported an improvement of oral mucosa and gingival wound healing by both PRP and PRF^32–34^. Both products support wound closure by modulation of the contraction of granulation tissue fibroblasts via altering the actin cytoskeleton^35^. We observed an inhibition of fibroblasts and osteoblasts migration by ZA, as previously reported^9^. In a recent study by Huang *et al*., concentrations between 10 and 15 μM were established as a threshold at which ZA inhibited migration of osteoblasts^36^. Although we used a higher concentration of ZA (80 μmol/l), we did not observe this side effect to this extent after adding PRP/PRF. In view of osteoblast cell culture, a positive effect on migration was observed, especially by PRF administration. PRP seemed to have a less positive effect on osteoblast migration. The results closely match those obtained by previous studies that observed the relatively limited effect of PRP on bone regeneration^37–39^. This can be attributed to the faster growth factor release from PRP, compared to the slow but constant release in PRF^37^. Normally, PRF has a natural fibrin scaffold that can protect growth factors from proteolysis, so that growth factors can maintain their activity over a longer period of time^40^. In our study, we used lysates of PRP and PRF according to a previous protocol. Although both lysates were used in a liquid form, we observed an enhancement of migration in the PRF group. Hence, a further factor, apart from the fibrin scaffold, seems to play a reasonable role to improve the proliferative and migrative properties of PRF in comparison to PRP in osteoblasts cell culture.

A simple explanation of the different effects of PRP and PRF on osteoblasts based on the preparation method cannot be reliably provided. Activation of PRP by addition of thrombin, which is known to have a negative effect on osteoblast migration, has not been used in the present study^41^.

The number of platelets cannot provide an explanation for the different effects of the two platelets derivatives since the PRP used in this study was produced from a higher number of platelets compared to PRF. Nevertheless, there are some differences in the production steps of PRF and PRP that may lead to these results. For instance, for PRP preparation we used anticoagulative substance, a leukocyte depletion filter and chose a lower PRP concentration (2.5%) than PRF (5%). Related studies have shown that different concentrations of PRP and PRF may show different biological effects^42,43^. Choi *et al*. and Yamada *et al*. showed that any concentration below or above 2–5% of PRP seems to inhibit cell proliferation and osteogenic effects^21–23^. On contrast, comparable studies have recommended the use of 20% exudate as an effective dilution of PRP and PRF to improve cell proliferation and migration *in vitro*^37,44^. The rise in RTCA measured cell index during the first 12 h observed in the current study appears to reflect cell adherence to the chambers. Afterwards PRF showed a steady increase of osteoblast and fibroblast proliferation in contrast to PRP. This result may indicate the positive influence of leukocytes on cell proliferation and migration in PRF^45^. Leukocytes seem to increase GF release of TGF-beta1, VEGF, PDGF-AB and contribute to angiogenesis, matrix production and hypercellularity^46,47^. Related studies have shown an advantage in wound healing using leukocyte enriched PRP products in a dose-dependent manner, too^46,48–51^. The use of ZA in this study led to a decrease of both osteoblasts and fibroblasts proliferations as previously proved in comparable investigations^36,52^. Interestingly, we observed a decrease in this effect on cell viability when adding PRF to ZA in both cell lines, especially in fibroblast culture. Considering osteoblasts, we observed an enhancement in cell viability only within 24 h using PRP. Similar results have been described in a previous study investigating osteoblast viability in PRP- and PRF-enriched culture, suggesting that PRF enhanced migration and proliferation of human osteoblasts more relatively to PRP^53^. Within the limitations of an *in vitro* study, the encouraging results presented here support the hypotheses that PRF and PRP may enhance bone healing in ZA-treated patients with osteonecrosis of the jaw. Furthermore, this study had shown additionally an advantage of PRF compared to PRP. Further experimental and clinical studies are warranted to assess the clinical benefit of local application of PRF or PRP in patients with bisphosphonate-associated osteonecrosis of the jaw.

## Material and Methods

### Ethics statement

Human blood studies were conducted according to the principles expressed in the Declaration of Helsinki and after receiving the approval of the Ethic Committee of the University Luebeck (ID 16–348). All subjects provided informed written consent for the collection of samples and subsequent analysis.

### Preparation of PRP

PRP was prepared using the buffy coat method routinely performed in blood banks to produce pooled platelet concentrates containing at least 2 × 10^11^ platelets^54^. Whole blood collected from health volunteers was first centrifuged for 20 min at 22 °C and 3931 g (630RS Centrifuge; Rotator 4292-B; Hettich, Kirchlengern Germany), to obtain the buffy coat. Afterwards, four buffy coat units and one plasma unit (32% plasma and 68% storage solution for platelets) from four donors with identical blood group types were pooled. After a second centrifugation for 17 min at 22 °C and 237 g (630RS Centrifuge; Rotator 4292-B; Hettich, Kirchlengern Germany), the platelet concentrate was put into a storage bag via a leukocyte depletion filter. In the next step, the PRP was incubated for increase of growth factors release by Freeze-Thaw-Freeze cycles^55–58^. After incubation, centrifugation was performed at 2000 g (Alegra X-12R-Centrifuge, Rotor SX 4750-A; Beckman-Coulter, Brea USA) for 20 min at 18 °C. Platelet-released supernatants were then frozen and maintained at −80 °C until experiments were performed.

### Preparation of PRF

Blood samples were taken from three healthy volunteers aged between 20–40 years to produce PRF. Each volunteer donated 10 ml of blood. Centrifugation was carried out at 400 g (Alegra X-12R-Centrifuge, Rotor SX 4750-A; Beckman-Coulter, Brea USA) for 10 min at 18 °C. Afterwards, the plasma and the buffy coat/fibrin clot were removed from the tubes and the rest was discarded. The growth factor release of PRF was increased using 37 °C incubation method according to Kobayashi *et al*.^59^. Finally, it was centrifuged for 20 min at 18 °C and 2000 g (Alegra X-12R-Centrifuge, Rotor SX 4750-A; Beckman-Coulter, Brea USA). The supernatant was transferred into a new tube and frozen at −80 °C until experiments were performed. The variation in the release of growth factors in both PRP and PRF depends on the number of platelets, the duration and performance of centrifugation, the incubation method and the presence of leukocytes^60,61^. The concentration of growth factors of this preparation method was investigated in a previous study^20^.

### Cells and cell culture conditions

Primary human gingival fibroblasts (hGF) and human osteoblasts (hOB) were purchased from Provitro® (Berlin, Germany). The cells were routinely cultured at 37 °C/5% CO_2_ and kept in the recommended growth medium until reaching the required number of cells. The fibroblast and osteoblast growth medium (FGM and OGM; both from Provitro®; Berlin Germany) were each supplemented with 10% fetal bovine serum (FBS, Provitro® GmbH) and 1% antibiotics (100 U/ml penicillin G, 100 μg/ml streptomycin). Experiments were performed with hGF and hOB between passages 3 and 10 in triplicate. Trypsin/EDTA solution (Biochrom®, Berlin, Germany) was used for passaging monolayer cultures.

### Preinvestigation for determination of optimal PRP and PRF concentration

A pre-test was performed with different concentrations of PRP and PRF to optimize their effect on cell proliferation. For this purpose a dilution series with 50%, 25%, 10%, 5% and 2.5% of PRP and PRF for the culture medium was prepared and the influence on cell proliferation was evaluated using the RTCA assay.

### Experimental design

PRP was used for cell culture experiments at a final concentration of 2.5% in the culture medium, whereas PRF was used at a concentration of 5%. The concentrations of 2.5% PRP and 5% PRF were chosen based on the results of the preinvestigation, showing that these concentrations are ideal for related cell proliferation. The concentrations chosen are conistent with those given by Choi *et al*. and Fernandes *et al*.^21–23^. Zoledronate (ZA; Zoledronsäure HEXAL® 4 mg/5 ml, Holzkirchen, Germany) was obtained in sterile 4 mg/5 ml intravenous infusion form. ZA was diluted with cell culture medium to obtain a concentration of 80 μmol/l. The ZA concentration used in culture media was based on the usual therapeutic dosage^62^. While the plasma concentration of ZA is approximately 1 μg/ml (3 × 10^−6^ M), concentrations in bone increase by 100-fold shortly after infusion^63^. Accordingly, the concentration used was 80 μmol/l. For each assay, the cells were cultivated and assessed for 72 h following the addition of seven different supplements: (I) negative control (NC): OGM or FGM + 1% FBS, (II) positive control (PC): OGM or FGM + 10% FBS (manufacture recommended medium), (III) PRP 2.5% + NC, (IV) PRF 5% + NC, (V) ZA (80 μmol/l) + NC, (VI) ZA (80 μmol/l) + PRP 2.5% + NC and (VII) ZA (80 μmol/l) + PRF 5% + NC.

### MTT assay

The kit used quantifies viable cells based on the mitochondrial conversion of 3-(4,5-dimethylthiazol-2-yl)-2,5-diphenyltetrazoliumbromid (MTT). Approximately 5 to 10 × 10^3^ cells were dispensed in a 96-well plate and cultivated with different supplements. Afterwards, 10 µl of MTT dye (5 mg/ml) was added to each well and incubated for 1 h. The crystals were then dissolved and gently shaken for 24 h at room temperature. The absorption of the reduced formazan product in the control and experimental wells was measured using a multiwell ELISA reader at a wavelength of 570 and 690 nm.

### Scratch assay

Cell migration was analyzed using IBIDI culture inserts (IBIDI®, Martinsried, Germany). The cells were seeded into culture insert plates at a concentration of 1.75 × 10^5^ cells per culture well for gingival fibroblasts and 2 × 10^5^ cells per culture well for osteoblasts. When the cells reached 100% confluence after 24 h of incubation, the culture inserts were removed. Cell debris in the wells was removed by washing gently with PBS before replacing the medium with different supplements. Photographs of the migration of the cells into the scratch area were taken every 5–12 h using a Zeiss Axiovert 200 M microscope (Carl Zeiss®, Jena, Germany). They were taken until closure of the scratch area and up to 7 days after removing the inserts. The area was determined using the ImageJ software ecosystem Fiji (National Institutes of Health, Bethesda, USA)^36^ and the MRI Wound Healing Tool plug-in.

### Proliferation assay using a real-time cell analyzer (RTCA)

The RTCA instrument (xCELLigence RTCA DP instrument; Roche Diagnostics GmbH, Mannheim, Germany) was used to analyze the proliferation properties of gingival fibroblasts and osteoblasts with the different experimental approaches. Based on preliminary results, 10 × 10^3^ osteoblasts and 5 × 10^3^ fibroblasts were used. Initially, 100 µl of cell-free growth medium was added to the wells of a 16x microtiter plate (E-Plate, Roche Diagnostics GmbH, Mannheim, Germany) using different supplements. After 30 min, the background impedance was measured for each well. Subsequently, 50 µl of the cell suspension with the desired number of cells was added to each well. According to the manufacturer’s guidelines, the plate was left at RT for 30 min to allow cell adhesion before being locked into the RTCA DP device incubator. The adhesion, spread and proliferation of the cells were monitored in 15 min intervals. The experiment was carried out for 72 h. The impedance of the cell sensor was described and measured as the cell index (CI). The CI value at each time point is defined as Rn–Rb/Rb, where Rn is the cell electrode impedance of the well and Rb is the background impedance of the well alone with the medium.

### Statistical evaluation

For verification, each experiment was performed in triplicate. The statistical evaluation was carried out with the statistical package IBM SPSS Statistics Version 24 (IBM, Stadt, Land). Data are expressed as median and confidence interval (CI) of 95%. Results were evaluated by Kruskal-Wallis-H and Mann-Whitney-U-test. A *p*-value of *p* ≤ 0.05 was considered statistically significant.
